# Evidence for neuroprotection by *Bacillus coagulans* ATCC 7050 via synaptic plasticity and oxidative balance in Alzheimer’s disease

**DOI:** 10.1038/s41598-025-28207-y

**Published:** 2025-12-29

**Authors:** Mohammad Saremi, Samaneh Safari, Mohammad Yousef Alikhani, Alireza Komaki, Seyed Davar Siadat, Babak Asghari

**Affiliations:** 1 Department of Microbiology, School of Medicine, Hamadan University of Medical Sciences, Hamadan, Iran; 2 Department of Neuroscience, School of Advanced Medical Sciences and Technologies, Hamadan University of Medical Sciences, Hamadan, Iran; 3 Infectious Disease Research Center, Avicenna Institute of Clinical Sciences, Hamadan University of Medical Sciences, Hamadan, Iran; 4 Department of Mycobacteriology and Pulmonary Research, Pasteur Institute of Iran, Tehran, Iran; 5 Microbiology Research Center (MRC), Pasteur Institute of Iran, Tehran, Iran

**Keywords:** Alzheimer’s disease, Bacillus coagulans, Long-term potentiation, Oxidative stress, Working memory, Biochemistry, Neurology, Neuroscience

## Abstract

Alzheimer’s disease (AD) is a progressive neurodegenerative disorder characterized by cognitive decline, synaptic impairment, and oxidative stress. Probiotics with antioxidant and anti-inflammatory properties have been proposed as potential adjunctive strategies. This study examined whether oral administration of *Bacillus coagulans* ATCC 7050 could attenuate hippocampal oxidative stress, modulate synaptic plasticity, and influence spatial working memory in an Aβ_1–42_-induced rat model of AD. Adult male Wistar rats were assigned to Sham, AD, BC (probiotic only), and AD + BC groups. Working memory was assessed by Y-maze, synaptic function by perforant path–dentate gyrus long-term potentiation (LTP) recordings, and oxidative status by hippocampal malondialdehyde (MDA), superoxide dismutase (SOD), and glutathione peroxidase (GPx) assays. AD rats exhibited reduced alternation percentage, impaired LTP (fEPSP slope and PS amplitude), elevated MDA, and decreased SOD and GPx activities versus Sham. *B. coagulans* treatment improved alternation percentage without affecting total entries, preserved PS amplitude from 30 min post-HFS, reduced MDA, and restored SOD activity, with partial GPx recovery. fEPSP slope remained reduced. These findings suggest *B. coagulans* ATCC 7050 mitigates oxidative stress, preserves neuronal excitability, and improves working memory in an Aβ-based AD model, supporting further investigation of its potential as a safe adjunct in early-stage disease.

## Introduction

Alzheimer’s disease (AD) is a progressive neurodegenerative disorder and the leading cause of dementia, affecting over 55 million people worldwide as of recent estimates^1^. It is characterized by gradual cognitive decline, particularly impairments in memory and learning, and hallmark pathologies such as extracellular deposition of amyloid-β (Aβ) peptides, intracellular neurofibrillary tangles composed of hyperphosphorylated tau, and synaptic degeneration^1–3^. While the etiology of AD is multifactorial, mounting evidence implicates oxidative stress and synaptic dysfunction as central contributors to early-stage cognitive decline^4,5^.

The hippocampus is particularly vulnerable in early AD due to its high density of Aβ-sensitive synapses and pivotal role in memory consolidation^6^. Soluble Aβ oligomers interfere with synaptic transmission and plasticity mechanisms such as long-term potentiation (LTP), even prior to substantial neurodegeneration. These alterations manifest as reductions in field excitatory postsynaptic potential (fEPSP) slope and population spike (PS) amplitude^7,8^.

Oxidative damage appears to underlie many of these effects: Aβ impairs mitochondrial function, elevates reactive oxygen species (ROS), and leads to lipid peroxidation and protein/DNA oxidation^9,10^. Concurrently, key antioxidant enzymes such as superoxide dismutase (SOD) and glutathione peroxidase (GPx) are often reduced in AD brains, which may further impair redox-sensitive signaling essential for synaptic maintenance^11,12^.

Probiotics have gained attention as adjunct therapeutic candidates due to their antioxidant, anti-inflammatory, and microbiota-modulating properties and several strains have demonstrated improved cognitive or molecular outcomes in AD models^13–15^. Among probiotic candidates, *Bacillus coagulans* is a spore-forming, heat-stable bacterium capable of surviving the gastrointestinal tract and producing riboflavin, short-chain fatty acids, and other bioactive metabolites with potential neuroprotective functions^16,17^. Certain strains also influence cytokine profiles and redox homeostasis^18,19^, while modulating gut microbial communities linked to neuroimmune regulation^19,20^. The *Bacillus coagulans* ATCC 7050 strain was selected due to its demonstrated high riboflavin production, antioxidant capabilities, gut microbiota modulatory effects, and documented immunomodulatory properties that support neuroprotection^16,17,21,22^.

Despite preliminary evidence for its antioxidant and immunomodulatory properties^20,23^, the impact of *B. coagulans* on electrophysiological and redox markers in AD brain tissue remains unclear. In this study, we investigated whether oral administration of *B. coagulans* ATCC 7050 could alleviate hippocampal oxidative stress, enhance synaptic plasticity, and improve hippocampal-dependent spatial working memory in a rat model of AD induced by intracerebroventricular injection of Aβ_1–42_.

## Results

### *Bacillus coagulans* ATCC 7050 attenuates Aβ_1–42_-induced working memory deficits

**Fig. 1:**
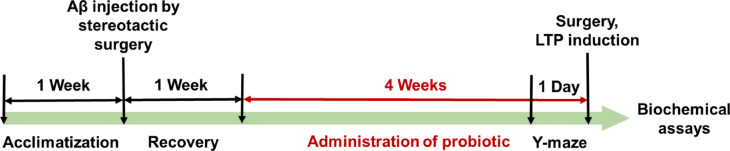
Experimental design. Schematic representation of the experimental timeline. Alzheimer’s disease was induced by intracerebroventricular injection of Aβ_1–42_. Oral administration of *Bacillus coagulans* ATCC 7050 was initiated a week after Aβ injection and continued daily for 4 weeks. Behavioral testing (Y-maze), electrophysiological recordings (perforant path–dentate gyrus LTP), and biochemical assays (MDA, SOD, GPx) were performed at the end of the treatment period.

**Fig. 2:**
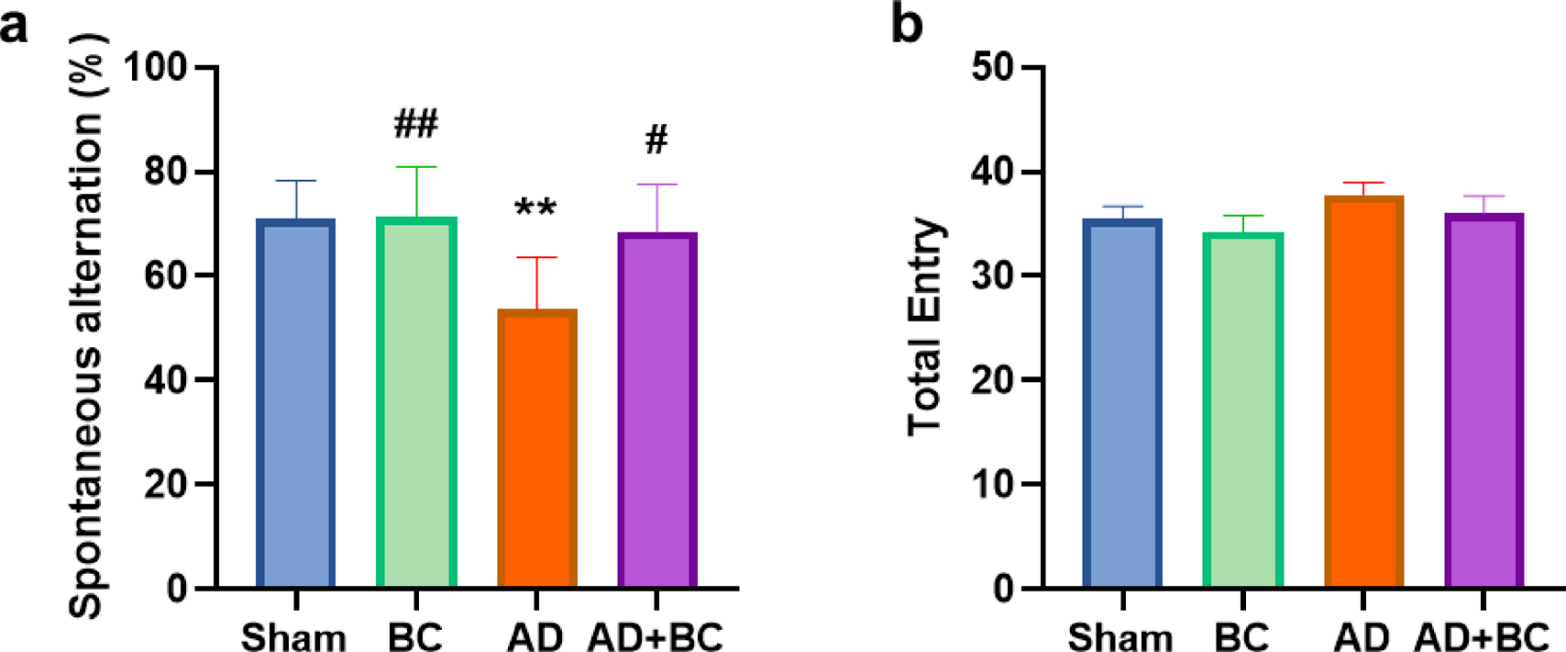
Effect of *Bacillus coagulans* ATCC 7050 treatment on working memory and locomotor activity in Aβ_1–42_-injected rats evaluated by Y-maze test. (**a**) Spontaneous alternation percentage in the Y-maze as an index of spatial working memory. (**b**) Total arm entries in the Y-maze as an index of locomotor activity. Rats were assigned to Sham, Alzheimer’s disease (AD), *B. coagulans* only (BC), and AD treated with *B. coagulans* (AD + BC) groups (*n* = 8 per group). ** *P* < 0.01 vs. Sham; # *P* < 0.05, ## *P* < 0.01 vs. AD. All values are expressed as mean ± SEM.

The experimental design is illustrated in Fig. 1. Working memory, assessed by spontaneous alternation percentage in the Y-maze, differed significantly among groups (F(3,28) = 6.826, *P* = 0.0014). The AD group showed a significantly lower alternation percentage compared with Sham (*P* = 0.0036; Fig. 2a) and BC (*P* = 0.003). Oral administration of *B. coagulans* ATCC 7050 in AD rats (AD + BC) significantly increased alternation percentage relative to AD (*P* = 0.0152), reaching values comparable to Sham and BC groups (*P* > 0.05 for both). No significant differences were observed between Sham and BC.

Total arm entries, an index of locomotor activity, did not differ significantly among groups (F(3,28) = 1.027, *P* = 0.3957), with no pairwise comparisons reaching statistical significance (all *P* > 0.05; Fig. 2b). This indicates that group differences in alternation percentage reflected cognitive rather than motor effects.

### *Bacillus coagulans* ATCC 7050 preserves PS amplitude but not fEPSP slope in Aβ_1–42_-injected rats

**Fig. 3:**
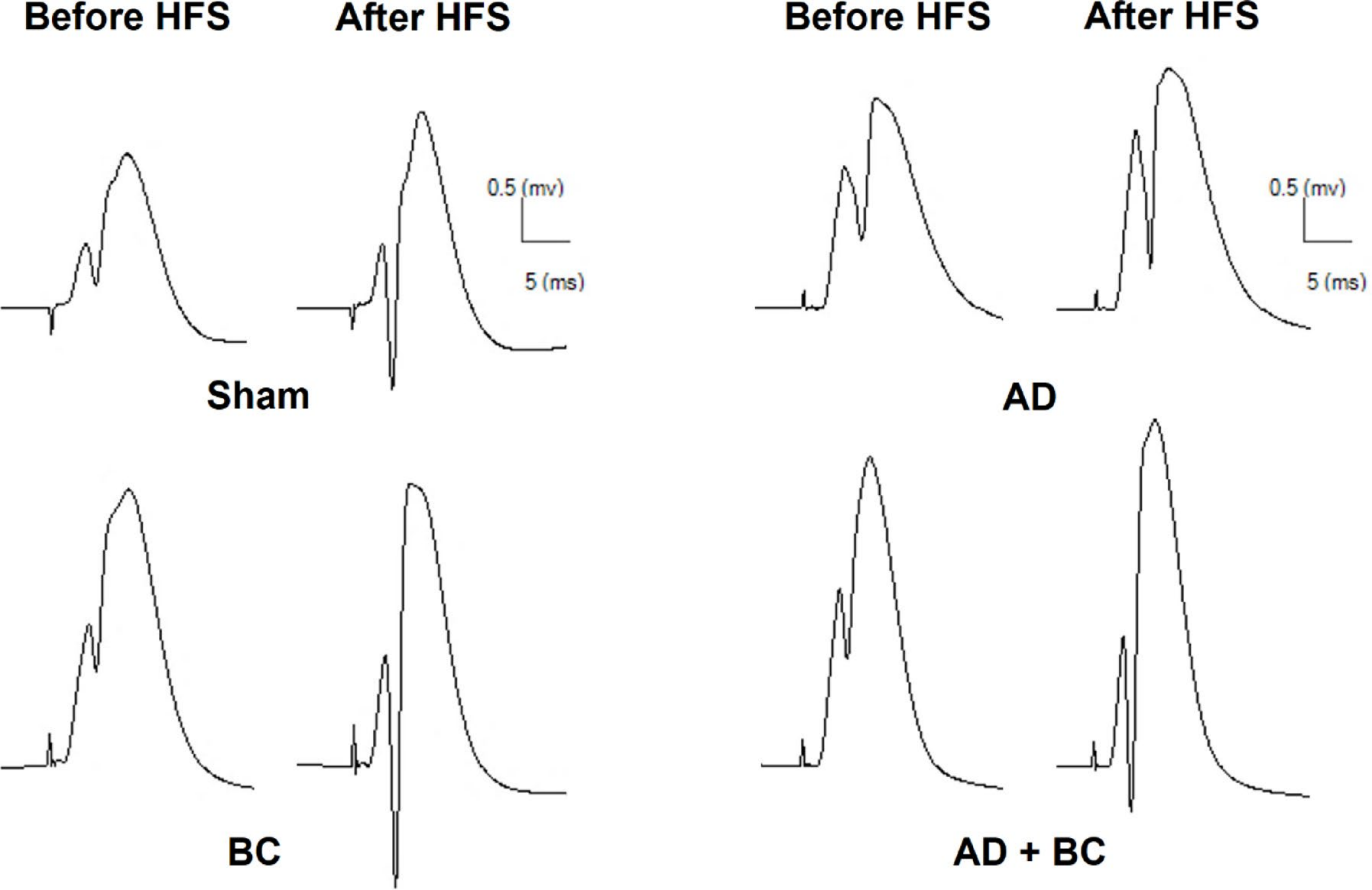
Sample traces of evoked field potentials in the dentate gyrus (DG) recorded before and 5 min after high-frequency stimulation (HFS) of the perforant pathway (PP) in all experimental groups.

**Fig. 4:**
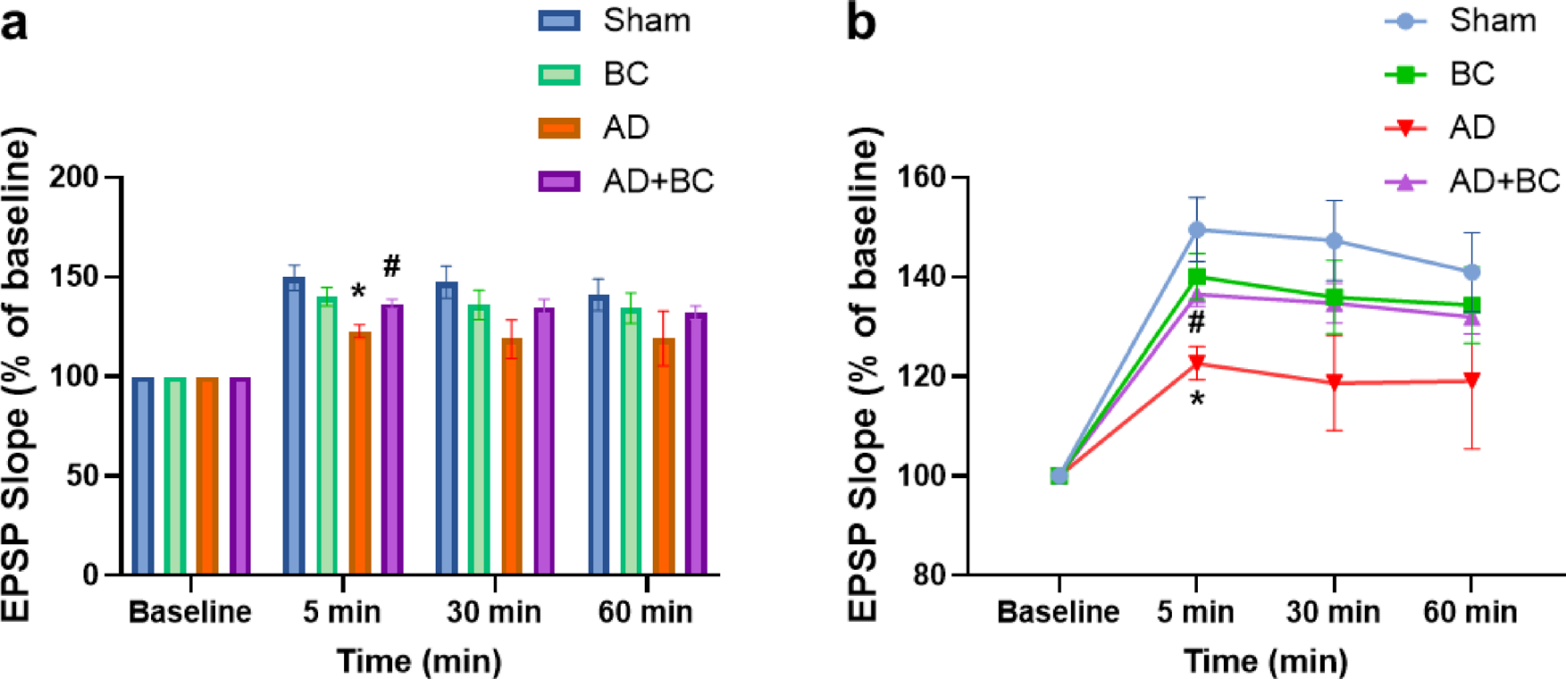
Effect of *Bacillus coagulans* ATCC 7050 on fEPSP slope in the dentate gyrus of Aβ_1–42_-injected rats. (**a**) Changes in fEPSP slope before and after high-frequency stimulation (HFS). (**b**) Time course of fEPSP slope expressed as a percentage of baseline over 60 min following HFS (*n* = 6 per group). * *P* < 0.05 vs. Sham; # *P* < 0.05 vs. AD (two-way repeated-measures ANOVA followed by Tukey’s post hoc test). All values are expressed as mean ± SEM.

Representative traces recorded from the dentate gyrus before and 5 min after high-frequency stimulation (HFS) are shown in Fig. 3. For fEPSP slope, two-way repeated measures ANOVA revealed a significant main effect of time (F(1.558, 31.16) = 51.17, *P* < 0.0001), but not group (F(3, 20) = 2.954, *P* = 0.0573), and no significant time × group interaction (F(9, 60) = 1.281, *P* = 0.266). In the AD group, potentiation at 5 min post-HFS was significantly reduced compared with Sham (*P* = 0.0274; Fig. 4) and remained impaired throughout the recording period. Although the elevation of fEPSP slope in the AD + BC group compared to AD at the 5-minute mark was statistically significant (*P* = 0.033), this effect was transient and not sustained at later time points, suggesting short-term synaptic facilitation rather than stable long-term potentiation.

**Fig. 5:**
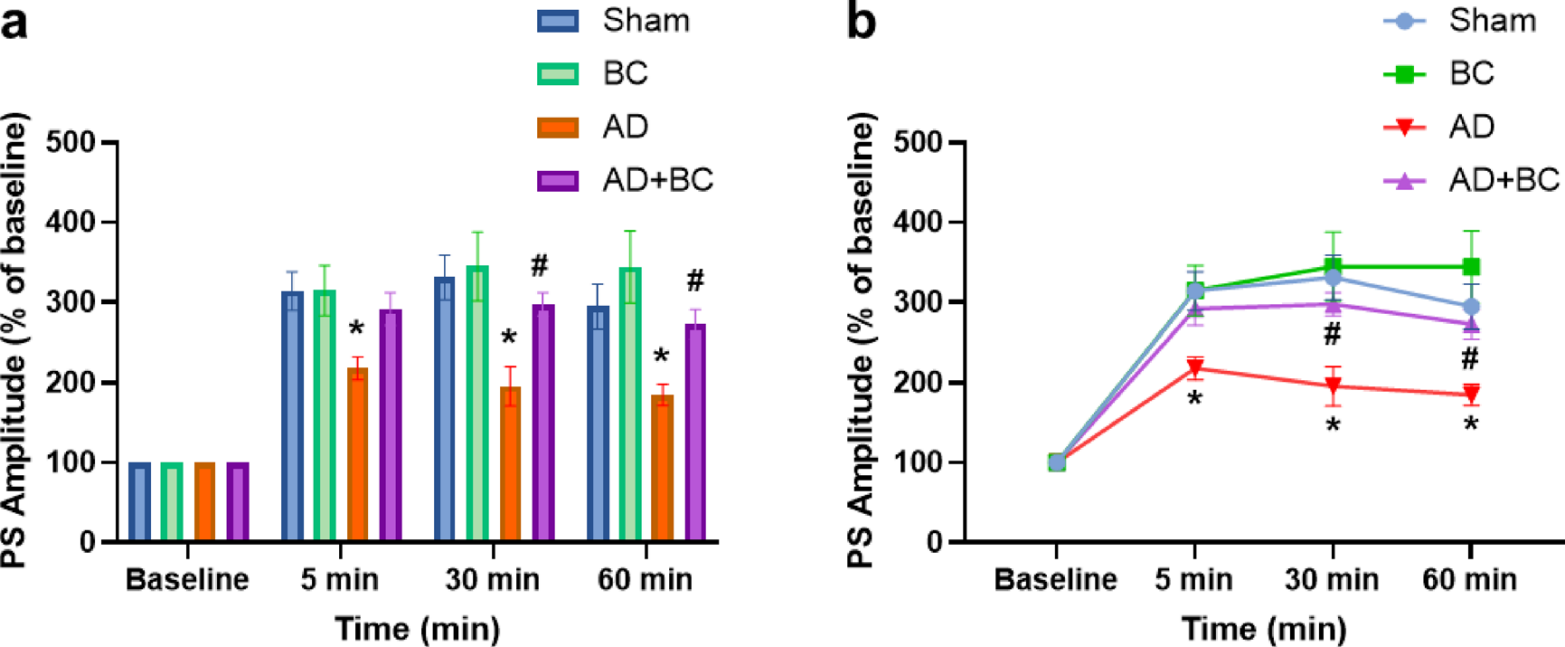
Effect of *Bacillus coagulans* ATCC 7050 on population spike (PS) amplitude in the dentate gyrus of Aβ_1–42_-injected rats. (**a**) Changes in PS amplitude before and after high-frequency stimulation (HFS) across Sham, AD, BC, or AD + BC groups (*n* = 6 per group). (**b**) Time course of PS amplitude expressed as percentage of baseline over 60 min post-HFS. * *P* < 0.05 vs. Sham; # *P* < 0.05 vs. AD (two-way repeated-measures ANOVA followed by Tukey’s post hoc test). All values are expressed as mean ± SEM.

PS amplitude showed a distinct pattern, with significant main effects of time (F(2.028, 40.56) = 125.6, *P* < 0.0001) and group (F(3, 20) = 5.548, *P* = 0.0062), and a significant interaction (F(9, 60) = 3.893, *P* = 0.0006). The AD group displayed reduced PS amplitude compared with Sham at 5 min (*P* = 0.0343; Fig. 5), 30 min (*P* = 0.0211), and 60 min (*P* = 0.0368) post-HFS. In AD + BC, PS amplitude was significantly higher than AD at 30 min (*P* = 0.0309) and 60 min (*P* = 0.0166). BC alone did not differ from Sham in either fEPSP slope or PS amplitude.

### *Bacillus coagulans* ATCC 7050 reduces hippocampal lipid peroxidation in Aβ_1–42_-injected rats

**Fig. 6:**
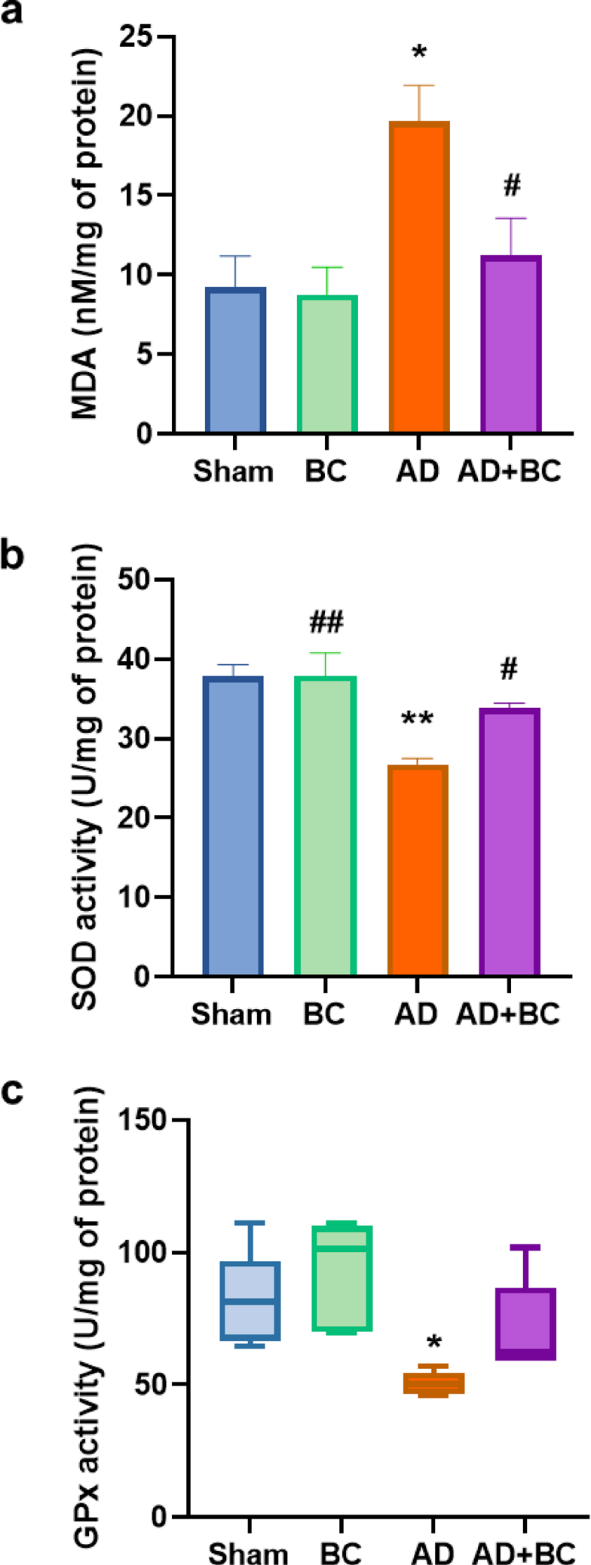
Effect of *Bacillus coagulans* ATCC 7050 treatment on hippocampal oxidative stress markers in Aβ_1–42_-injected rats. (**a**) Malondialdehyde (MDA) levels in the hippocampus (lipid peroxidation marker) shown as mean ± SEM. (**b**) Superoxide dismutase (SOD) activity, expressed as mean ± SEM. (**c**) Glutathione peroxidase (GPx) activity, shown as median and interquartile range (IQR) using box plot; statistical analysis performed with Kruskal–Wallis test and Dunn’s post hoc test due to non-normal distribution of GPx data. Rats were assigned to Sham, BC, AD, or AD + BC groups (*n* = 5 per group). * *P* < 0.05, ** *P* < 0.01 vs. Sham; # *P* < 0.05, ## *P* < 0.01 vs. AD.

Hippocampal malondialdehyde (MDA) levels differed significantly among groups (F(3, 16) = 5.954, *P* = 0.0063; Fig. 6a). AD rats showed significantly elevated MDA compared with Sham (*P* = 0.0127), consistent with increased lipid peroxidation. This elevation was significantly attenuated in AD + BC compared with AD (*P* = 0.0493; Cohen’s d = 1.65, η² = 0.53), indicating a large effect size despite the borderline p-value, whereas BC alone did not differ from Sham (*P* = 0.9988).

### Superoxide dismutase activity is preserved and glutathione peroxidase partially protected by *Bacillus coagulans* ATCC 7050 in AD model rats

SOD activity was significantly lower in the AD group compared with Sham (F(3,16) = 9.679, *P* = 0.0007; Fig. 6b). Post hoc analysis showed that SOD activity was significantly reduced in AD compared with both Sham (*P* = 0.0014) and BC (*P* = 0.0013) groups. In AD + BC, SOD activity was significantly higher than in AD (*P* = 0.0418) and was not different from Sham, indicating restoration to near-normal levels.

GPx activity also differed significantly among groups (Kruskal–Wallis statistic = 12.58, *P* = 0.0056; Fig. 6c). GPx activity was significantly reduced in AD compared with Sham (*P* = 0.0277) and BC (*P* = 0.0067). Although GPx activity was numerically higher in the AD + BC group compared to AD, this difference was not statistically significant (*P* = 0.3259), suggesting only a partial recovery or potentially the need for a longer treatment duration. No significant differences in GPx activity were observed between Sham and BC.

## Discussion

This study demonstrates that oral administration of *Bacillus coagulans* ATCC 7050 partially restores hippocampal synaptic plasticity, improves working memory, and rebalances redox homeostasis in a rat model of Alzheimer’s disease (AD) induced by intracerebroventricular injection of Aβ_1–42_. While full recovery of long-term potentiation (LTP) was not achieved, the observed improvements in population spike (PS) amplitude, working memory, and antioxidant enzyme activity suggest that *B. coagulans* confers neuroprotective effects, likely mediated through modulation of oxidative stress and neuronal network excitability^18,23^.

In the AD group, high-frequency stimulation produced a blunted LTP response—reflected by reduced fEPSP slope and PS amplitude—consistent with established Aβ-induced synaptic dysfunction. The reduction in fEPSP slope at 5 min post-HFS suggests early-phase LTP impairment, possibly due to disrupted NMDA receptor activation or postsynaptic signaling^24^, in line with prior Aβ injection studies showing concurrent synaptic transmission deficits and oxidative stress^8,25^. While *B. coagulans* treatment did not significantly improve fEPSP slope, PS amplitude—reflecting synchronous neuronal firing influenced by intrinsic excitability and inhibitory tone—was significantly improved at later time points. This suggests preferential stabilization of postsynaptic spiking activity or enhancement of network-level synchrony, underscoring the complexity of probiotic effects across electrophysiological compartments.

Several potential mechanisms may underlie this fEPSP–PS dissociation. The probiotic may preferentially modulate postsynaptic excitability rather than presynaptic glutamate release, potentially through (1) modulation of local GABAergic inhibitory tone, which regulates spike threshold in dentate gyrus granule cells, (2) alterations in voltage-gated ion channels critical for action potential generation, or (3) enhanced postsynaptic receptor expression or signaling cascades^25,26^. Similar fEPSP–PS dissociations have been observed with interventions that selectively modulate intrinsic excitability or inhibitory circuits without altering basal synaptic transmission^25,26^. Future studies employing paired-pulse ratios or direct measurements of inhibitory currents would help clarify these mechanisms.

The electrophysiological recovery was accompanied by significant improvement in Y-maze spontaneous alternation without changes in total arm entries, indicating enhanced working memory rather than altered locomotor activity. This behavioral outcome supports the functional relevance of PS amplitude recovery, consistent with reports that interventions restoring LTP also enhance performance in hippocampal-dependent memory tasks^27–29^. Previous works have shown that probiotics can improve spatial learning, short-term working memory, and long-term retention in AD models^13,14,29,30^, and *B. coagulans* JA845 improved Morris water maze and passive avoidance performance in D-Galactose/AlCl_3_-induced mice^23^.

At the biochemical level, our results reveal that *B. coagulans* treatment counteracts hippocampal oxidative stress in Aβ-injected rats. The AD group showed significantly elevated MDA levels—a marker of lipid peroxidation—and reduced activities of key antioxidant enzymes, superoxide dismutase (SOD) and glutathione peroxidase (GPx). These redox disturbances are well documented in AD pathology and closely tied to mitochondrial dysfunction and ROS generation^31^. Notably, *B. coagulans* supplementation significantly reduced MDA levels and restored SOD activity, with a partial increase in GPx activity. This pattern mirrors findings that certain *B. coagulans* strains activate Nrf2-related pathways and upregulate antioxidant enzymes^23,32–34^. The association between improved oxidative balance, PS amplitude, and working memory supports the role of redox regulation in maintaining synaptic function. In addition, prior reports show *B. coagulans* strains attenuate neuroinflammation via modulation of cytokine pathways^23,33^, which could further safeguard synaptic integrity.

The antioxidative effects observed here are consistent with known metabolic capabilities of *B. coagulans*. For the ATCC 7050 strain specifically, production of riboflavin (vitamin B₂) may support glutathione recycling via its role as a cofactor for glutathione reductase^16,17^. Riboflavin has also been shown to reduce mitochondrial oxidative stress and suppress inflammasome activation by inhibiting caspase-1 and lowering pro-inflammatory cytokine release^16^. In addition, related *B. coagulans* strains produce short-chain fatty acids, exopolysaccharides with radical-scavenging activity, and bacteriocins, all of which may modulate oxidative stress and inflammation^18,33^. *B. coagulans* supplementation has also been reported to influence the gut–brain axis by promoting beneficial taxa and reducing pro-inflammatory endotoxin-producing bacteria^20,33,34^. Such changes could indirectly support hippocampal function by lowering systemic inflammation and enhancing neurotrophic signaling. While metabolites and microbiota composition were not assessed in this study, they remain plausible contributors to the observed neuroprotection; future work should include targeted metabolomic, microbiota, and inflammatory profiling to clarify these mechanisms.

Limitations include the absence of full fEPSP slope recovery, suggesting that *B. coagulans* alone may not fully restore dendritic potentiation and could require combination therapies targeting glutamatergic signaling. Lack of cytokine and microbiota analysis limits mechanistic resolution, and the use of only male rats precludes assessment of sex-specific responses.

## Conclusion

In conclusion, *Bacillus coagulans* ATCC 7050 alleviated hippocampal oxidative damage, partially restored synaptic plasticity, and improved working memory in an Aβ-based AD model. These findings support the potential of *B. coagulans* as a safe adjunctive approach to mitigate early synaptic and oxidative disturbances in AD, warranting further studies integrating behavioral, molecular, and microbiota analyses to define active mechanisms.

## Methods

### Animal ethics

All procedures were approved by the Ethics Committee of Hamadan University of Medical Sciences (IR.UMSHA.AEC.1403.016) and conducted in accordance with the National Institutes of Health Guide for the Care and Use of Laboratory Animals. All efforts were made to minimize stress and suffering. This study is reported in accordance with the ARRIVE guidelines (https://arriveguidelines.org).

### Probiotic preparation

Spray-dried spores of *Bacillus coagulans* ATCC 7050 formulated in dextrose powder (1 × 10¹⁰ spores/g) were generously provided by Pardis Roshd Mehregan Co. (Shiraz, Iran). To verify the viable spore concentration, 1 gram of the probiotic powder was suspended in 10 mL of distilled water, resulting in a final concentration of 1 × 10⁹ spores/mL. The suspension was heat-treated at 80 °C for 15 min to eliminate any remaining vegetative cells and spore counts were confirmed by surface plating after tenfold serial dilution.

### Experimental design

Adult male Wistar rats (250–290 g) were obtained from the animal facility of Hamadan University of Medical Sciences and housed under standard conditions (23 ± 2 °C; 12:12 h light/dark cycle) with *ad libitum* access to food and tap water. All animals were acclimatized for one week prior to experimentation.

Animals were randomly divided into four groups (*n* = 8 per group):

1. Sham-operated group (Sham): Receiving the oral administration of distilled water containing 0.1% dextrose after right lateral ventricle injection of phosphate-buffered saline (PBS) as the solvent of Aβ_1–42_ for 4 weeks.
2. Alzheimer’s model group (AD): Receiving the oral administration of distilled water containing 0.1% dextrose after AD induction for 4 weeks.
3. *Bacillus coagulans*-treated group (BC): Sham-operated animals receiving the oral administration of the spore suspension (1 × 10⁹ spores/mL, 1 mL/day) by oral gavage for 4 weeks.
4. Alzheimer’s disease + *Bacillus coagulans*-treated group (AD + BC): Receiving the oral administration of the spore suspension (1 × 10⁹ spores/mL, 1 mL/day) by oral gavage after AD induction for 4 weeks.

All experimental groups began with *n* = 8 animals per group. For electrophysiological recordings, 2 animals per group failed to establish stable baseline recordings (signal-to-noise ratio < 3:1, unstable field potentials) and were excluded prior to data analysis based on pre-defined quality control criteria applied blinded to group allocation, resulting in *n* = 6 for LTP analysis. For biochemical assays, one animal per group yielded insufficient hippocampal tissue for complete biochemical replicates (MDA, SOD, GPx), resulting in *n* = 5 per group.

### Preparation and administration of Aβ_1–42_

Aβ_1–42_ peptide (500 µg; SCP0038, Sigma-Aldrich, Germany) was dissolved in 500 µL sterile PBS and incubated at 37 °C for 7 days to allow neurotoxic fibril formation. Fibrillar formation was confirmed according to the protocol used by Komaki et al. (2019) using identical incubation conditions, previously validated to yield neurotoxic fibril species with consistent bioactivity^8^. To induce an Alzheimer’s disease (AD) model, rats were anesthetized with an intraperitoneal injection of ketamine (100 mg/kg) and xylazine (10 mg/kg), then placed in a stereotaxic apparatus (Stoelting Co., Wood Dale, IL, USA). After securing the head and identifying the Bregma and Lambda coordinates, a small cranial hole was drilled over the right lateral ventricle using standard stereotaxic coordinates: anteroposterior (AP): −0.8 mm, mediolateral (ML): 1.4 mm, and dorsoventral (DV): 4.0 mm from the skull surface, based on the Paxinos and Watson rat brain atlas (2006). A total volume of 5 µL of the Aβ_1–42_ solution was slowly injected unilaterally into the right lateral ventricle using a 5-µL Hamilton microsyringe (Hamilton Laboratory Products, Reno, NV, USA) with a stainless-steel cannula. The injection was performed over 5 min (1 µL/min), and the needle was left in place for an additional 5 min to ensure adequate diffusion and prevent reflux^8^.

Sham-operated animals received the same volume of sterile PBS instead of the Aβ_1–42_ solution. Following the procedure, the scalp was sutured, and animals were transferred to individual cages with free access to food and water. The animals were allowed a recovery period of 7 days before the initiation of subsequent experimental interventions.

### Y‑maze spontaneous alternation test

Spatial working memory was assessed using a Y-maze consisting of three identical opaque Plexiglas arms (50 cm long, 15 cm wide, 35 cm high) arranged at 120° angles. Each animal was placed in the center of the apparatus and allowed to explore freely for 8 min. Spontaneous alternation was defined as consecutive entries into all three arms, and calculated as^14,35^:

$$Spontaneous~alternation~\left( \% \right)=\frac{{Number~of~alternations}}{{Total~entries - 2}}~ \times 100$$

The total number of entries was used as an index of locomotor activity. The maze was cleaned with 75% ethanol between trials.

The Y-maze spontaneous alternation test was selected because it assesses hippocampal-dependent working memory without requiring training or repeated exposures, thereby avoiding potential confounding effects on synaptic plasticity measurements.

### Electrophysiological recording and LTP induction

Electrophysiological procedures were adapted from previously established methods with minor modifications^8,26^. Briefly, rats were anesthetized via intraperitoneal injection of urethane (1.5 g/kg; Sigma-Aldrich, USA) and placed in a stereotaxic apparatus (Stoelting Co., Wood Dale, IL, USA). Core body temperature was maintained at 36.5 ± 0.5 °C using a regulated heating pad.

Following exposure of the skull, small burr holes were drilled for electrode placement. A concentric bipolar stainless-steel stimulating electrode (Teflon-coated, 125 μm diameter; Advent Co., UK) was implanted in the lateral perforant path (PP) at the following stereotaxic coordinates: AP: −8.1 mm from bregma; ML: +4.3 mm from midline; DV: 3.2 mm from the skull surface. A similar bipolar recording electrode was placed in the dentate gyrus (DG) granule cell layer at AP: −3.8 mm, ML: +2.3 mm, and DV: 2.7–3.2 mm, based on the Paxinos and Watson rat brain atlas. To minimize cortical trauma, electrodes were lowered slowly (0.2 mm/min), and optimal placement was verified via real-time electrophysiological monitoring of field potentials evoked by single-pulse PP stimulation.

To determine stimulation intensity, an input-output profile was obtained by varying the amplitude of single 0.1 ms biphasic square-wave pulses (delivered at 0.1 Hz) and averaging 10 responses per level. The final stimulus intensity was adjusted to evoke approximately 40% of the maximal population spike (PS) amplitude. Test stimuli were then applied every 10 s, and baseline recordings were acquired for 35–40 min to ensure signal stability.

Long-term potentiation (LTP) was induced via a high-frequency stimulation (HFS) protocol consisting of 10 bursts of 20 stimuli at 400 Hz, with 0.2 ms pulse duration and 10-second interburst intervals. The stimulation protocol was delivered using a programmable isolator unit (A365, World Precision Instruments, USA), and signals were amplified (1000×) and filtered (1 Hz to 3 kHz) using a differential amplifier (DAM 80, WPI, USA). Responses were digitized at 10 kHz sampling rate and visualized via eTrace software (ScienceBeam, Iran) for further analysis.

All surgical and electrophysiological procedures were conducted by experienced personnel using established protocols. No animals died during intracerebroventricular injection of Aβ_1–42_ or during in vivo electrophysiological recording procedures.

### Measurement of evoked potentials

The evoked field potential in the DG was assessed by two key components: the slope of the field excitatory postsynaptic potential (fEPSP) and the amplitude of the population spike (PS).

The fEPSP slope was calculated between 20% and 80% of the rising phase of the initial positive deflection, using the formula:

$$\:EPSP\:=\frac{\varDelta\:V}{\varDelta\:T}$$

where ΔV is the change in voltage over the selected portion of the rising phase, and ΔT is the corresponding change in time.

The PS amplitude was calculated as:

$$PS=\frac{{\Delta V1+\Delta V2}}{2}$$

where ΔV_1_ and ΔV_2_ represent the voltage differences from the first positive peak to each of the subsequent two negative peaks in the waveform.

### Biochemical analyses

At the end of the electrophysiological recordings, euthanasia was performed by ketamine/xylazine overdose, in accordance with institutional approval and the AVMA Guidelines for the Euthanasia of Animals. Hippocampi were isolated and stored at − 80 °C. The right hippocampus was used for all biochemical analyses. Tissues were homogenized in ice-cold phosphate buffer, centrifuged (12,000×g, 15 min, 4 °C), and supernatants used for analysis.

Superoxide dismutase (SOD) and glutathione peroxidase (GPx) activities were measured using colorimetric assay kits (ZellBio GmbH, Germany), following manufacturer instructions. Malondialdehyde (MDA) levels were determined by thiobarbituric acid reactive substances (TBARS) assay, as an index of lipid peroxidation using commercial colorimetric kits (ZellBio GmbH, Germany). All values were normalized to total protein content determined by the Bradford method.

### Statistical analysis

Data are expressed as mean ± SEM and analyzed in GraphPad Prism^®^ v.10. Normality was assessed with the Shapiro–Wilk test. For normally distributed data, two-way repeated measures ANOVA with Tukey’s post hoc test was used for LTP, and one-way ANOVA with Tukey’s post hoc test was used for Y-maze, SOD, and MDA. For non-normal data (GPx), the Kruskal–Wallis test with Dunn’s post hoc test was applied.

To quantify LTP, the slope of the field excitatory postsynaptic potential (fEPSP) or the amplitude of the population spike (PS) recorded at each time point following high-frequency stimulation (HFS) was normalized to its baseline value using the following formula:

$$\:\mathrm{L}\mathrm{T}\mathrm{P}\:\left(\mathrm{\%}\right)=\frac{EPSP\:or\:PS\:value\:after\:HFS\:induction}{Average\:EPSP\:or\:PS\:at\:baseline}\times\:100\%$$

Significance was set at *p* < 0.05.

## Data Availability

All data supporting the findings of this study are available within the article. Raw datasets are available from the corresponding author on reasonable request.
